# *ChCDC25* Regulates Infection-Related Morphogenesis and Pathogenicity of the Crucifer Anthracnose Fungus *Colletotrichum higginsianum*

**DOI:** 10.3389/fmicb.2020.00763

**Published:** 2020-05-08

**Authors:** Yaqin Yan, Jintian Tang, Qinfeng Yuan, Qiongnan Gu, Hao Liu, Junbin Huang, Tom Hsiang, Lu Zheng

**Affiliations:** ^1^The Key Lab of Plant Pathology of Hubei Province, Huazhong Agricultural University, Wuhan, China; ^2^Zhejiang Provincial Key Laboratory of Biometrology and Inspection & Quarantine, College of Life Sciences, China Jiliang University, Hangzhou, China; ^3^Institute of Plant Protection and Soil Science, Hubei Academy of Agricultural Sciences, Wuhan, China; ^4^School of Environmental Sciences, University of Guelph, Guelph, ON, Canada

**Keywords:** *Colletotrichum higginsianum*, *ChCDC25*, appressorial formation, cAMP signaling pathway, pathogenicity

## Abstract

The fungal pathogen, *Colletotrichum higginsianum*, causes a disease called anthracnose on various cruciferous plants. Here, we characterized a *Saccharomyces cerevisiae CDC*25 ortholog in *C. higginsianum*, named *ChCDC25* (CH063_04363). The *ChCDC25* deletion mutants were defective in mycelial growth, conidiation, conidial germination, appressorial formation, and invasive hyphal growth on *Arabidopsis* leaves, resulting in loss of virulence. Furthermore, deletion of *ChCDC25* led to increased sensitivity to cell wall stress and resulted in resistance to osmotic stress. Exogenous cyclic adenosine monophosphate (cAMP) and IBMX treatments were able to induce appressorial formation in the *ChCDC25* mutants, but abnormal germ tubes were still formed. The results implied that *ChCDC25* is involved in pathogenicity by regulation of cAMP signaling pathways in *C. higginsianum*. More importantly, we found that ChCDC25 may interact with Ras2 and affects Ras2 protein abundance in *C. higginsianum*. Taken together, *ChCDC25* regulates infection-related morphogenesis and pathogenicity of *C. higginsianum.* This is the first report to reveal functions of a *CDC*25 ortholog in a hemibiotrophic phytopathogen.

## Introduction

*Colletotrichum higginsianum* is an important hemibiotrophic fungal pathogen that causes anthracnose disease on various cruciferous plants (Narusaka et al., 2004; O’Connell et al., 2004). The pathosystem involving *C. higginsianum* and *Arabidopsis thaliana* is an attractive model to examine fungal–plant interactions because both components have had their genomes sequence, because of the interesting infection strategy by the pathogen (Yan et al., 2018). On the host plant, a set of specialized infection structures is generated by the fungus, consisting of germ tubes, appressoria, biotrophic hyphae, and necrotrophic hyphae (O’Connell et al., 2012).

Ras proteins regulate various signaling networks including cellular processes, morphogenesis, differentiation, and polar growth (Jiang et al., 2018). In plant pathogenic fungi, Ras proteins are well known as playing a key role in pathogenicity (Turrà et al., 2014). In *Magnaporthe oryzae*, expression of the dominant active MoRAS2^*G*18*V*^ construct in transformants leads to abnormal appressorial formation and the loss of pathogenicity (Zhou et al., 2014). Similar to *M. oryzae*, BcRAS1 has been verified to be involved in growth, morphogenesis, and pathogenicity in *Botrytis cinerea* (Dub et al., 2013). Bluhm et al. (2007) demonstrated that Ras proteins switch between active (GTP-bound) and inactive (GDP-bound) states to regulate signal transduction cascades. Ras guanine exchange factors (RasGEFs) regulate the active state, whereas Ras GTPase-activating proteins (RasGAPs) regulate the inactive state of Ras proteins (Shields et al., 2000). When the balance between these states is disturbed, abnormal growth in the fungus may occur.

In *Saccharomyces cerevisiae*, CDC25 acts as a RasGEF in regulation of Ras proteins to affect cell cycle, growth, and proliferation (Broach, 1991; Boy-Marcotte et al., 1996). In *Candida albicans*, lack of a RasGAP, IRA2, resulted in defective filamentous growth and more sensitivity to heat stress, whereas *CDC25* knockout mutants were less filamentous than normal (Enloe et al., 2000). In *Colletotrichum orbiculare*, CoIra1, a GAP, plays a critical role in conidial germination, appressorial formation, appressorial penetration, and pathogenicity by regulating cyclic adenosine monophosphate (cAMP)–PKA and MAPK signaling pathways through inactivation of CoRas2 (Harata and Yasuyuki, 2014). In *Ustilago maydis*, the CDC25 homolog Sql2 is an activator of Ras2, dispensable for vegetative growth but required for pathogenic development (Müller et al., 2003). However, the role of CDC25 homologs in hemibiotrophic phytopathogens is still unknown.

In this study, we characterized an *S. cerevisiae CDC25* ortholog in *C. higginsianum*, named *ChCDC25*. To explore gene function of *ChCDC25*, we generated *ChCDC25* deletion mutants and complementation transformants. Our results showed that ChCDC25 was localized in the cytoplasm and regulated vegetative growth, conidiation, appressorial formation, stress responses, and pathogenicity of *C. higginsianum*. In addition, our data suggested that ChCDC25 can interact especially with Ras2 and affected Ras2 protein abundance in *C. higginsianum*.

## Materials and Methods

### Strains, Plasmids, and Plants

*Colletotrichum higginsianum* strain Ch-1 was used as the wild-type strain (O’Connell et al., 2004). All fungal strains were cultured on potato dextrose agar (PDA) at 25°C in the dark. *A. thaliana* Col-0 plants were used in virulence assays.

### Knockout and Complementation of *ChCDC25*

To replace *ChCDC25*, approximately 1 kb of upstream and downstream flanking sequences of the gene were, respectively, amplified using the primer pairs Ch*CDC*252F1F/Ch*CDC*25F1R and Ch*CDC*25F2F/Ch*CDC*25F2R (Supplementary Table S1). The PCR products of the upstream and downstream flanking sequences were digested and ligated into the hph cassette released from pMD18T-HYG, resulting in the initial vector F1-HYG-F2. This vector was digested and ligated with pNeo3300III to construct gene replacement vector pNeo3300IIIChCDC25-KO. Fungal transformation was conducted with the *Agrobacterium tumefaciens*-mediated transformation (ATMT) protocol (Liu et al., 2013). Putative transformants were placed on PDA amended with 50 μg/mL hygromycin B (Roche, Mannheim, Germany). The putative knockout mutants with hygromycin B resistance were further confirmed by Southern blotting and reverse transcription–polymerase chain reaction (RT-PCR) analysis.

To perform a complementation assay, the region containing 1.5-kb upstream sequence with native promoter and full-length coding sequence of *ChCDC*25 without stop codon was cloned into green fluorescent protein vector pNeo3300-GFP. The EHA105 strain with the complementation vector was transformed into the ChCDC25 deletion mutant with ATMT. The transformants were selected on PDA supplemented with 150 μg/mL G418 (Ameresco, Cleveland, United States). The putative complementation mutants with G418 resistance were further confirmed by RT-PCR analysis. Primers used in amplification of the PCR products are shown in Supplementary Table S1.

### Phenotype Analysis

Mycelial plugs were transferred to PDA plates and incubated in darkness at 25°C. After 7 days, vegetative growth rate and conidial production were measured. Mycelia were collected from 7-day-old liquid cultures in potato dextrose broth (PDB), dried at 50°C, and weighed. Hyphae taken from the edge of colonies on PDA were examined for morphology of hyphal tips by light microscopy (Nikon, Tokyo, Japan). For appressorial formation assays, conidia were harvested by flooding PDA plates with sterile distilled water and gathered by passing through cheesecloth. The conidial suspension was adjusted to a concentration of 1 × 10^5^ spores/mL and placed onto plastic cover slips (Thermo Fisher Scientific, Waltham, United States) incubated in 100% RH at 25°C for 24 h. Conidial germination and appressorial formation rates were quantified microscopically. Exogenous cAMP and IBMX (3-isobutyl-1-methylxanthine) were added to conidial suspensions for final concentrations of 10 and 2.5 mM, respectively. Conidial germination and appressorial formation rates were determined by microscopy examining at least 100 conidia or appressoria per treatment. Each test was repeated three times.

### Stress Response

Osmotic, cell wall integrity, and oxidative stress assays were carried out on PDA plates amended with different chemicals, including 0.7 M NaCl, 1 M sorbitol (Sinopharm, Shanghai, China), 0.05% sodium dodecyl sulfate (SDS) (Sigma, Darmstadt, Germany), 0.25 mg/mL Congo red (CGR) (Yuanhang, Shanghai, China), or 4 mM H_2_O_2_ (Yuanhang). Inhibition rates were calculated based on the method of Gu et al. (2017). Each test was repeated three times.

### Quantification of Intracellular cAMP

Mycelia were harvested from 2-day-old PDB cultures, quickly frozen in liquid nitrogen, and lyophilized for 16 h. For each treatment, 10 mg of ground samples was resuspended in 200 mL ice-cold 6% trichloroacetic acid. After incubation on ice for 10 min, the samples were centrifuged at 4000 r/min for 15 min at 4°C. The supernatant was transferred and washed four times with water-saturated diethyl ether. The cAMP levels were determined using the cAMP Biotrak Immuno-assay System (Abbkine, Wuhan, China). The experiment was repeated three times.

### Pathogenicity and Plant Infection Assays

Because a small number of conidia were produced by ChCDC25 knockout mutants, pathogenicity tests were conducted by placing drops of 10 μL conidial suspension (1 × 10^5^ conidia/mL) on 4-week-old *A. thaliana* leaves. Inoculated plants were incubated in a dew chamber at 25°C in dark for 24 h and then transferred to a growth chamber in 12-h light/12-h dark. Disease lesions were observed at 4 days postinoculation (dpi). Wounding experiments were carried out on lightly pricked leaves, and droplets of 10 μL conidial suspension with 1 × 10^5^ conidia/mL were point-inoculated on wounded leaves. The plants were incubated in a dew chamber at 25°C, and symptoms were evaluated at 4 dpi. Each test was repeated three times.

To observe infection structures, inoculated leaves at 4 dpi were cleared in a solutions of methanol:chloroform:glacial acetic acid (6: 3: 1), stained with trypan blue, and viewed by microscopy.

### Nucleic Acid Manipulation, Quantitative RT-PCR, and RT-PCR

Vegetative hyphae were used to isolate genomic DNA with CTAB (Sambrook, 1989). To confirm deletion of *ChCDC*25, 15 mg *Sac*I-digested genomic DNA of the putative *ChCDC*25 deletion mutants was gel blotted onto Nylon + membrane (GE healthcare, Buckinghamshire, United Kingdom) and hybridized with a probe according to the manufacturer’s instruction (Amersham Gene Images Alkphos Direct Labeling and Detection System; GE Healthcare, Buckinghamshire, United Kingdom). Signals were detected using the ChemiDoc XRS + system (Bio-Rad, Hercules, CA, United States).

Gene expression levels of *ChCDC25* were assessed at different developmental stages: *C. higginsianum* mycelia grown for 3 days on PDB, conidia collected from 7-day-old PDA, and *Arabidopsis* leaves inoculated with Ch-1 and incubated for different intervals (24, 48, and 72 h). Total RNA was isolated with TRIzol Plus RNA Purification Kit (Invitrogen, Carlsbad, CA, United States), and RT assays were conducted with the TransScript One-Step gDNA Removal and cDNA Synthesis SuperMix (TransGen Biotech, Beijing, China) following manufacturer’s instructions. The relative gene expression levels were calculated by the 2^–Δ*Ct*^ method (Livak and Schmittgen, 2001). The *C. higginsianum* actin gene (CH063_01975) was used as the endogenous reference. For RT-PCR, the synthetic cDNA from transformants was used as template for knockout and complemented strain screening with specific primers (Supplementary Table S1).

### Subcellular Localization Analysis

For subcellular localization analysis of ChCDC25, complemented strains with strong GFP signal were selected for observation. GFP signals of mycelia, conidia, and appressoria (prepared as described above) were observed under a Zeiss LSM 510 Meta confocal microscope (Carl Zeiss, Jena, Germany).

### Yeast Two-Hybrid Assay

Full-length cDNA of *ChCDC25* was cloned into pGADT7 as the prey construct. The bait construct was generated by ligating cDNA of *ChRas1* or *ChRas2* into pGBKT7 (primers list in Supplementary Table S1). All constructs were confirmed by sequencing analysis and transformed in pairs into yeast strain Y2HGold (Takara, Dalian, China) with the Matchmaker^®^ Gold Yeast Two-Hybrid System (Takara, Dalian, China). The Trp + and Leu + transformants were isolated and assayed for growth on SD-Trp-Leu-His medium. To further investigate potential interaction domain, three domains of *ChCDC*25 were deleted separately for prey constructs. ChCDC25^Δ^ ^*SH*3^, ChCDC25^Δ^ ^*RasGEF_N*^, and ChCDC25^Δ^ ^*RasGEF*^ were individually cloned into prey vector pGADT7. The constructs were generated using in-fusion cloning (ClonExpress MultiS One Step Cloning Kit; Vazyme, Nanjing, China). The resulting prey and bait constructs of ChRas2 were introduced into Y2HGold in pairs. Yeast strains carrying pGBKT7-P53/pGADT7-T and pGBD/pGAD were used as positive and negative controls, respectively.

### Protein Extraction and Western Blotting

Total proteins were isolated from mycelia cultured in PDB for 3 days using a previously described method (Tang et al., 2020). For Western blotting, proteins were separated on a 12% SDS–polyacrylamide gel electrophoresis gel (Bio-Rad) and electrophoretically transferred to the polyvinylidene fluoride membrane (Millipore, Etobicoke, ON, Canada) in the Bio-Rad Mini Trans-Blot system. ChRas was detected with Anti-KRas Rabbit Monoclonal Antibody (Bioss, Beijing, China). Signals were captured by ChemiDoc XRS + system (Bio-Rad).

### Bioinformatics

The full sequence of *ChCDC*25 was downloaded from the *C. higginsianu*m genomic database^1^ of isolate Ch-1 (IMI349063). The protein domains were predicted using SMART^2^. Protein sequences of *CDC*25 homologs from different organisms were obtained using NCBI BLASTp. A phylogenetic tree was generated by MEGA7.0 with the neighbor-joining (NJ) algorithm using *S. cerevisiae* as the outgroup. The numbers at branch nodes are bootstrap percentages out of 1000 replicates.

### Statistical Analysis

The software SPSS 3.0 (SPSS Inc., Chicago, IL, United States) was used for analysis of variance of untransformed data. Upon finding a statistically significant effect, means were separated using least significant difference (LSD) at *P* = 0.05.

## Results

### Identification and Characterization of *ChCDC*25

**FIGURE 1:**
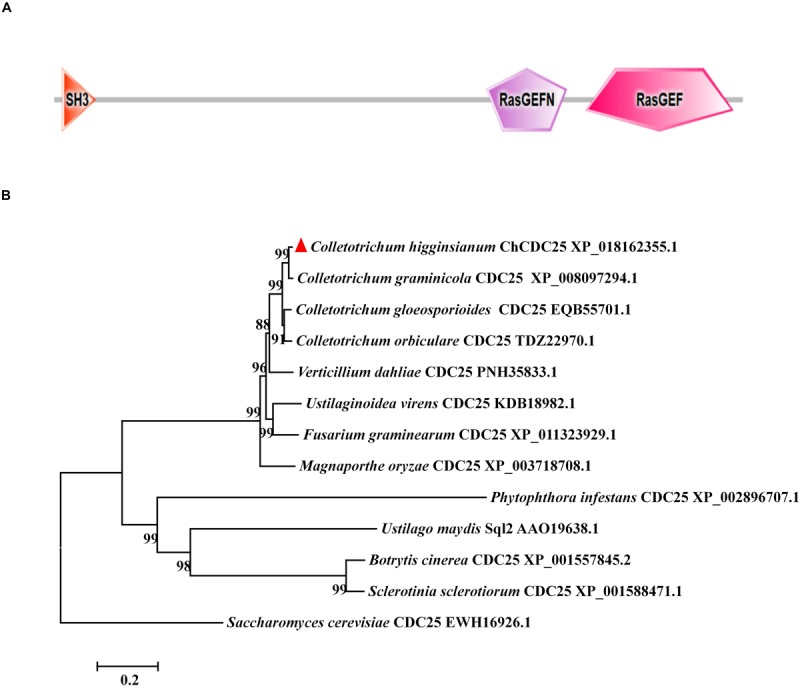
Conserved domain prediction and phylogenetic analysis of ChCDC25. **(A)** The domain structures of ChCDC25 as annotated by SMART (http://smart.embl-heidelberg.de). **(B)** NJ tree shows CDC25 in *C. higginsianum* and other selected fungi. Sequences were downloaded from the NCBI database, and their accession numbers are shown following the gene names. The numbers at branch nodes are bootstrap percentages out of 1000 replicates.

The CDC25 protein sequence (GenBank EWH16926.1) from *S. cerevisiae* was used to blast against the *C. higginsianum* genome database with BLASTp (*e* value > 1E-5). The results revealed that Ch063_04363 shared 44% amino acid sequence identity with *S. cerevisiae* CDC25, and we named it ChCDC25. Domain prediction with SMART showed that Ch063_04363 contained the same three domains as *S. cerevisiae* CDC25, including SH3, RasGEF, and RasGEFN (Figure 1A).

Phylogenetic analysis of CDC25 proteins revealed that CDC25 was highly conserved in *Colletotrichum* species, *Verticillium dahliae*, *Fusarium graminearum*, *Ustilaginoidea virens*, and *M. oryzae* (Figure 1B). However, identity levels of CDC25 proteins between *C. higginsianum* and other phytopathogens including *U. maydis*, *Sclerotinia sclerotiorum*, and *B. cinerea* were less than 20% (Figure 1B). These results indicated that CDC25 proteins are diverse among different plant pathogenic fungi.

### Deletion and Complementation of *ChCDC*25 in *C. higginsianum*

**FIGURE 2:**
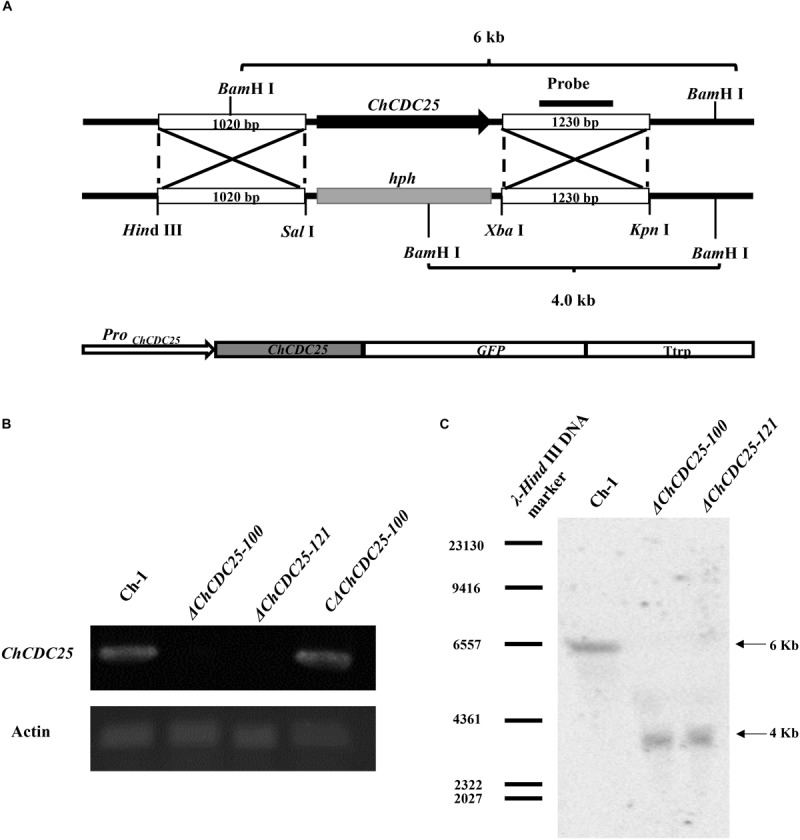
Targeted knockout and complementation of *ChCDC25*. **(A)** Strategic map construction of gene knockout and complementation vectors and sites for restriction enzymes. **(B)** Reverse transcription–PCR was used to check the expression of *ChCDC*25. Total RNA was extracted from mycelia of wild type, *ChCDC25-100*, *ChCDC25*-121, and C*ChCDC25-100*. **(C)** Confirmation of *ChCDC*25 deletion by Southern blotting. Genomic DNA was digested with *Bam*HI and hybridized with specific probes (probe indicated in **A**).

To determine gene function of Ch*CDC*25, *ChCDC*25 deletion mutants were generated by replacing the gene with *hph* gene cassette (Figure 2A). The putative *ChCDC*25 deletion mutants (Δ*ChCDC25-100* and Δ*ChCDC25-121*) were confirmed by RT-PCR and Southern blotting analysis (Figures 2B,C). Reverse transcriptase–PCR results showed that the *ChCDC*25 was only expressed in wild-type Ch-1 but not in deletion mutants Δ*ChCDC25-100* or Δ*ChCDC25-121* (Figure 2B). Southern blotting revealed that one 4.0-kb *Bam*HI band could be detected in the two *ChCDC*25 deletion mutants, which was shorter than that detected in Ch-1 (6.0 kb) (Figure 2C).

To determine whether the altered phenotypes in *ChCDC25* deletion mutants could be restored, a complementation assay was carried out with Δ*ChCDC25-100*. A *ChCDC*25-eGFP fusion construct with the native promoter was generated (Figure 2A) and then transformed into Δ*ChCDC25-100* by ATMT. Subsequently, the complementation transformant, CΔ*ChCDC25-100*, was confirmed by RT-PCR analysis, and *ChCDC*25 could be expressed in CΔ*ChCDC25-100* (Figure 2B).

### *ChCDC*25 Is Required for Vegetative Growth, Hyphal Morphology, and Conidiation

**FIGURE 3:**
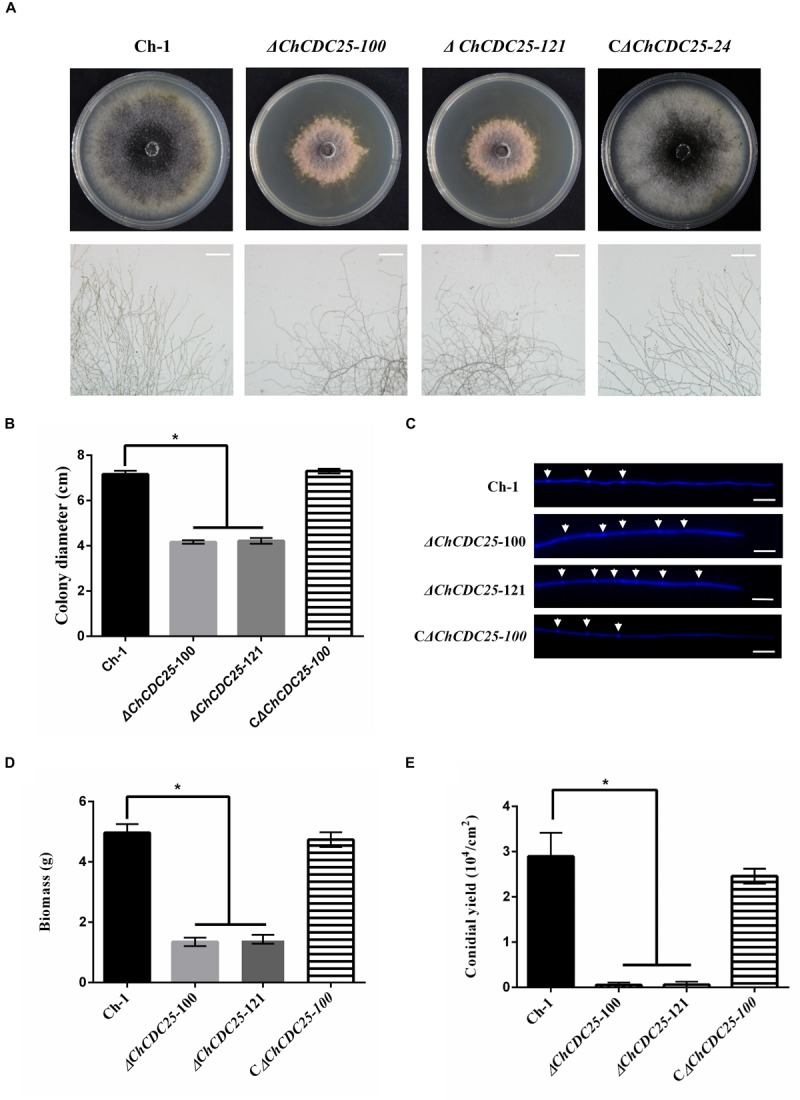
Colony morphology, mycelial tip morphology, colony growth rate, and conidiation of *ChCDC25* knockout mutants. **(A)** Morphology of colonies (upper) and mycelial tips (lower) of each strain. Ch-1 and mutants were grown on PDA for 7 days. Scale bar, 50 μm. **(B)** Statistical analysis of vegetative growth of Ch-1 and mutants. Colony diameter of each strain was measured after culturing on PDA at 25°C for 7 days. **(C)** Hyphal tips of Ch-1, *ChCDC25* mutants, and C*ChCDC25-100* were stained with Calcofluor White. The cell septa are indicated by white arrows. Scale bar, 20 μm. **(D)** Statistical analysis of mycelial biomass. **(E)** Statistical analysis of conidial production. There were three biological replicates for all treatments. Asterisks represent significant differences between the mutants and WT at *P* = 0.05.

After incubation on PDA, the *ChCDC25* deletion mutants formed a tightly compact colony, and colony diameters of ChCDC25 deletion mutants were significantly smaller than that of the wild type or the complemented strains (Figures 3A,B). Microscopic examination revealed that hyphal tips were radial and linear in the wild type, whereas hyphal tips were highly branched and distorted in ChCDC25 mutants (Figure 3A). In addition, by staining with CFW, more septa were found in apical hyphal compartments of *ChCDC25* deletion mutants (Figure 3C). The biomass in PDB was also measured, and *ChCDC25* deletion mutants displayed reduced growth in the liquid medium (Figure 3D). Furthermore, conidial production was drastically reduced in *ChCDC25* deletion mutants (Figure 3E). Overall, these results indicated that ChCDC25 was essential for vegetative growth and conidiation in *C. higginsianum*.

### *ChCDC*25 Is Involved in Cell Wall Integrity and Osmotic and Oxidative Stress Responses

**FIGURE 4:**
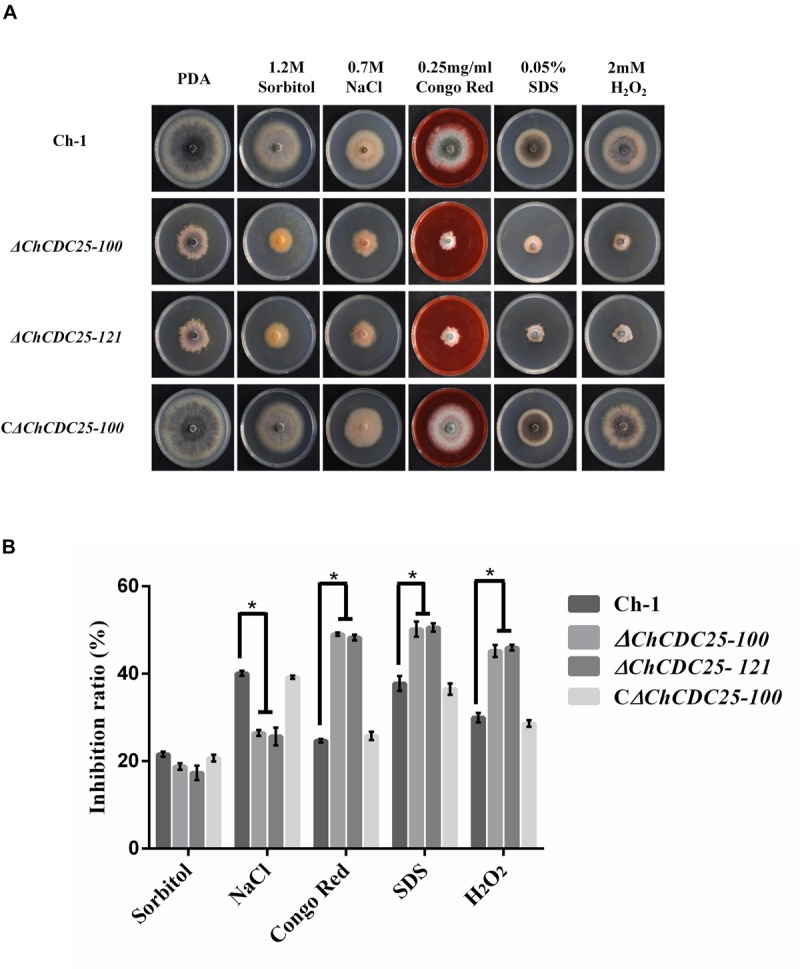
Stress responses of *ChCDC25* knockout mutants. **(A)** Colony morphology of Ch-1, *ChCDC25* knockout mutants, complementation strain on PDA supplied with different chemical stresses. All strains were cultured on PDA medium supplied with 1.2 M sorbitol, 0.7 M NaCl, 0.25 mg/mL Congo red, 0.05% SDS, or 4 mM H_2_O_2_. Cultures were photographed after 7 days at 25°C. **(B)** Inhibition rate calculated based on colony diameter of strains subjected to different stresses after 7 days. For each strain, the growth inhibition rate by different stresses was estimated in comparison with its growth on regular PDA. All treatments had three independent biological repeats. Asterisks represent significant differences between the mutant and WT at *P* = 0.05.

CDC25 plays an important role in stress response in *S. cerevisiae* (Folch-Mallol et al., 2004). However, the function of *ChCDC*25 in stress response remains unclear. Therefore, we assayed the growth defects of the *ChCDC25* deletion mutants on PDA amended with different stress chemicals (Figure 4A). Compared with the wild-type Ch-1 and the complementation transformant, the *ChCDC25* deletion mutants showed decreased tolerance to CGR, SDS, and H_2_O_2_ (Figure 4B), revealing that the deletion of *ChCDC*25 reduced the resistance of *C. higginsianum* to cell wall and oxidative stresses. On the other hand, the *ChCDC25* deletion mutants exhibited increased tolerance to NaCl compared to the wild-type Ch-1 (Figure 4B). These findings suggested that Ch*CDC*25 is involved in responses to hyperosmotic, cell wall, and oxidative stresses.

### *ChCDC*25 Is Involved in cAMP Signaling Pathways

**FIGURE 5:**
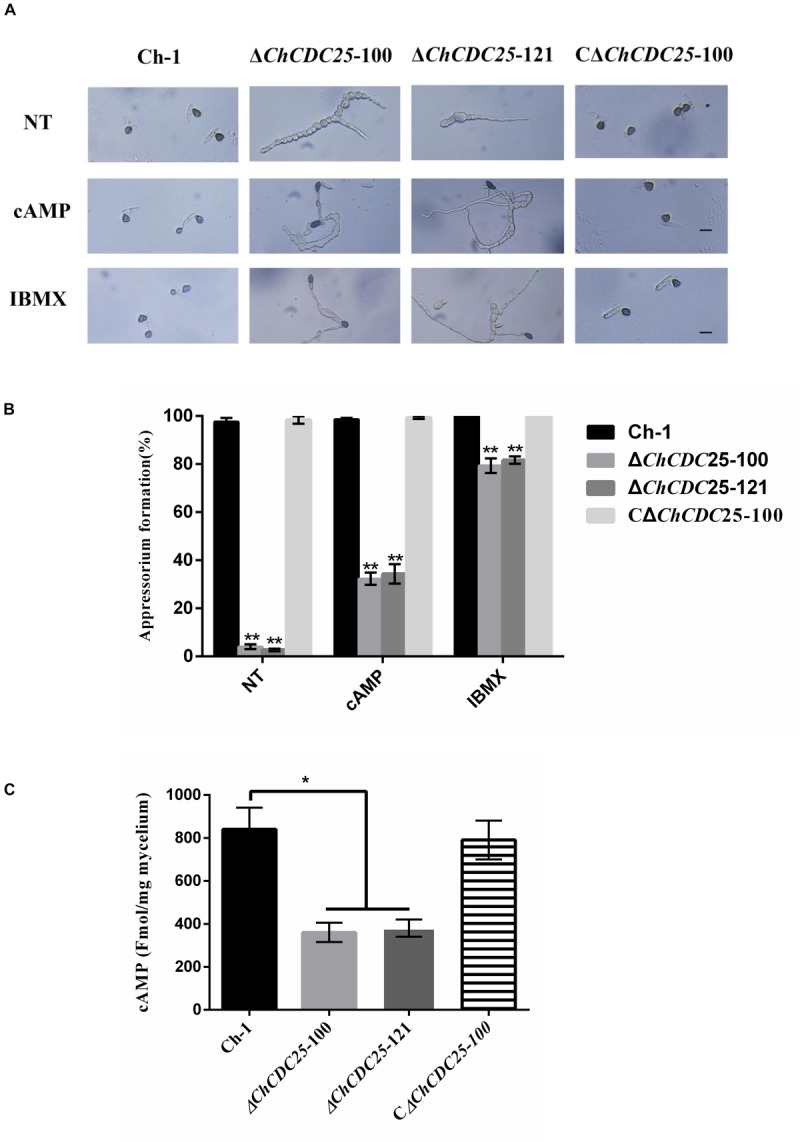
Appressorial formation regulated by *ChCDC*25 is functionally associated with the cAMP signaling pathway. **(A)** Appressorial formation of Ch-1, *ChCDC25* knockout mutants, and complementation strain on a hydrophobic surface or induced by 10 mM cAMP and 2.5 mM IBMX. Conidial suspensions of each strain in distilled water were incubated on a hydrophobic surface at 25°C for 24 h. NT (non-chemical treatment means treated with water other than a chemical). Scale bar, 10 μm. **(B)** Percentage appressorial formation of Ch-1 and mutants on hydrophobic surfaces. For each strain, at least 100 conidia were measured for every replicate. Three replicates were examined, and three independent experiments were conducted. Asterisks represent significant differences between the mutant and WT at *P* = 0.05. **(C)** Measurement of intracellular cAMP levels of mycelia grown in PDB.

Conidial germination and appressorial development of the strains were assessed on plastic coverslips to investigate infection-related morphogenesis. In the wild-type and the complementation strain, approximately 98% of conidia geminated and formed dark appressoria on hydrophobic surfaces by 24 h, but in *ChCDC25* deletion mutants, less than 5% of conidia could produce appressoria. In addition, germination tubes of ChCDC25 mutants were much longer than those of the wild-type strain and the complementation strain, indicating that ChCDC25 was involved in the surface recognition and appressorial formation (Figures 5A,B).

Appressoria are important infection structures that are regulated by cAMP signaling, and they have critical roles in *C. higginsianum*. Therefore, intracellular cAMP levels of the ChCDC25 deletion mutants were examined. Compared with the wild-type and complementation strains, cAMP level was reduced approximately 50% in the *ChCDC25* deletion mutants, suggesting that *ChCDC*25 might act upstream of the cAMP signaling pathway via cAMP biosynthesis regulation (Figure 5C).

To test whether appressorial formation of ChCDC25 deletion mutants was responsive to exogenous chemicals, cAMP and IBMX were added separately to conidial suspensions (Figure 5A). On hydrophobic surfaces, exogenous cAMP and IBMX could not restore full growth of germ tubes. However, cAMP could induce appressorial formation by *ChCDC25* deletion mutants, and approximately 80% of the conidia germinated, but efficiency of appressorial formation increased approximately only 30% by 24 h (Figure 5B). In contrast, under the same conditions, the appressorial formation rate of the *ChCDC25* deletion mutants induced by IBMX reached 80% (Figure 5B). These results indicated that *ChCDC*25 may be involved in cAMP signaling during appressorial formation in *C. higginsianum*.

### *ChCDC*25 Is Important for Virulence

**FIGURE 6:**
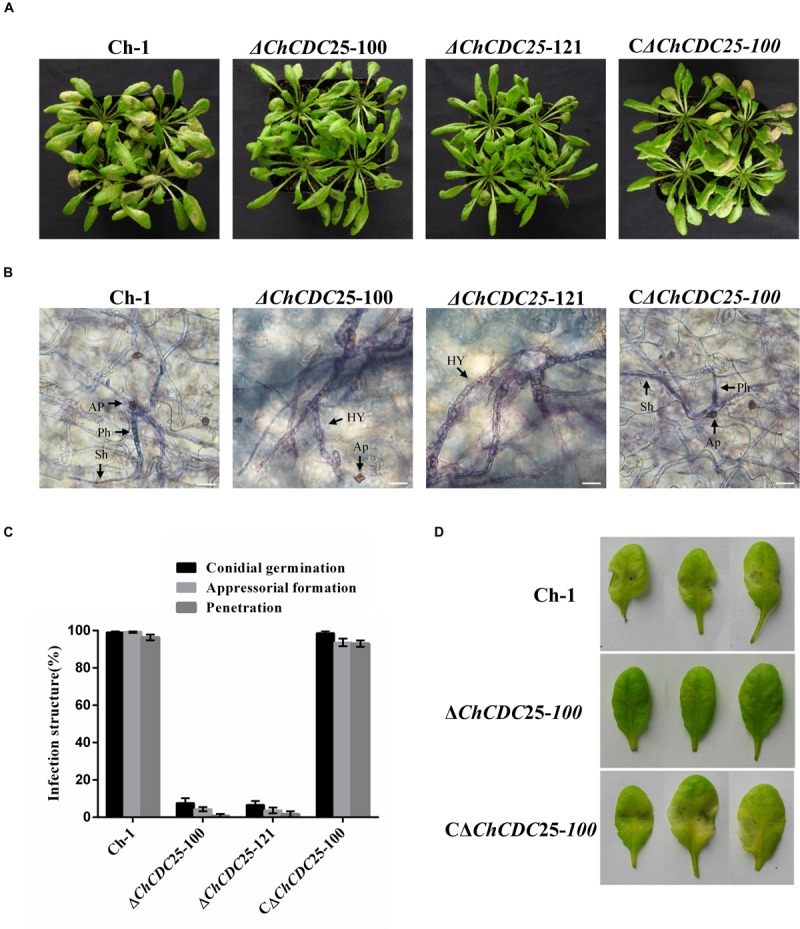
Pathogenicity and plant infection assays of *ChCDC25* knockout mutants on *Arabidopsis* leaves. **(A)** A 10 μL droplet of conidial suspension (1 × 10^5^ conidia/mL) placed each wounded *Arabidopsis* leaf and then incubated at 25°C for 4 days. **(B)** Infection structures in *Arabidopsis* leaf cells. Scale bar, 20 μm. Hy, hyphae; Ap, appressorium; Ph, primary hypha; Sh, secondary hypha. **(C)** Microscopic examination of fungal development in leaf tissues. For each strain, at least 100 conidia were measured for every replicate. Three replicates were examined and three independent experiments were conducted. **(D)** Pathogenicity assay of the *ChCDC25* mutants on wounded leaves. Conidial suspensions (10 μL) were placed on wound sites of *Arabidopsis* leaves and incubated at 25°C for 3 days.

To explore the role of *ChCDC*25 in pathogenicity, tests were conducted on the host plant. Because a small number of conidia were produced by ChCDC25 deletion mutants, conidial suspensions were spotted on *A. thaliana* leaves. *ChCDC25* deletion mutants failed to produce typical water-soaked lesions, whereas this defect was fully restored in the complemented strain (Figure 6A), indicating that *ChCDC*25 had an essential role in virulence of *C. higginsianum*.

To quantify the penetration rate of appressoria, inoculated leaves were stained with trypan blue and examined by microscopy at 4 dpi (Figure 6B). When biotrophic hyphae inside cells were observed, appressoria were considered to have penetrated successfully. At 4 dpi, both bulbous primary hyphae and thin, filamentous secondary hyphae of wild-type and complemented strains were formed and extensively colonized penetrated leaves. In contrast, only 3% of the appressoria of deletion mutants penetrated successfully (Figure 6C). These results suggest the *ChCDC25* deletion mutants were defective in conidial germination and appressorial formation on *A. thaliana* leaves, which was responsible for their loss of virulence.

To further test invasive growth ability of the ChCDC25 deletion mutants independent of penetration, conidial suspension of the strains was inoculated onto artificial wounds on *Arabidopsis* leaves. The *ChCDC25* deletion mutants barely caused any symptoms at the wounded sites after 3 days (Figure 6D), indicating that *ChCDC*25 was still required for invasive hyphal growth.

### Expression and Subcellular Localization of ChCDC25

**FIGURE 7:**
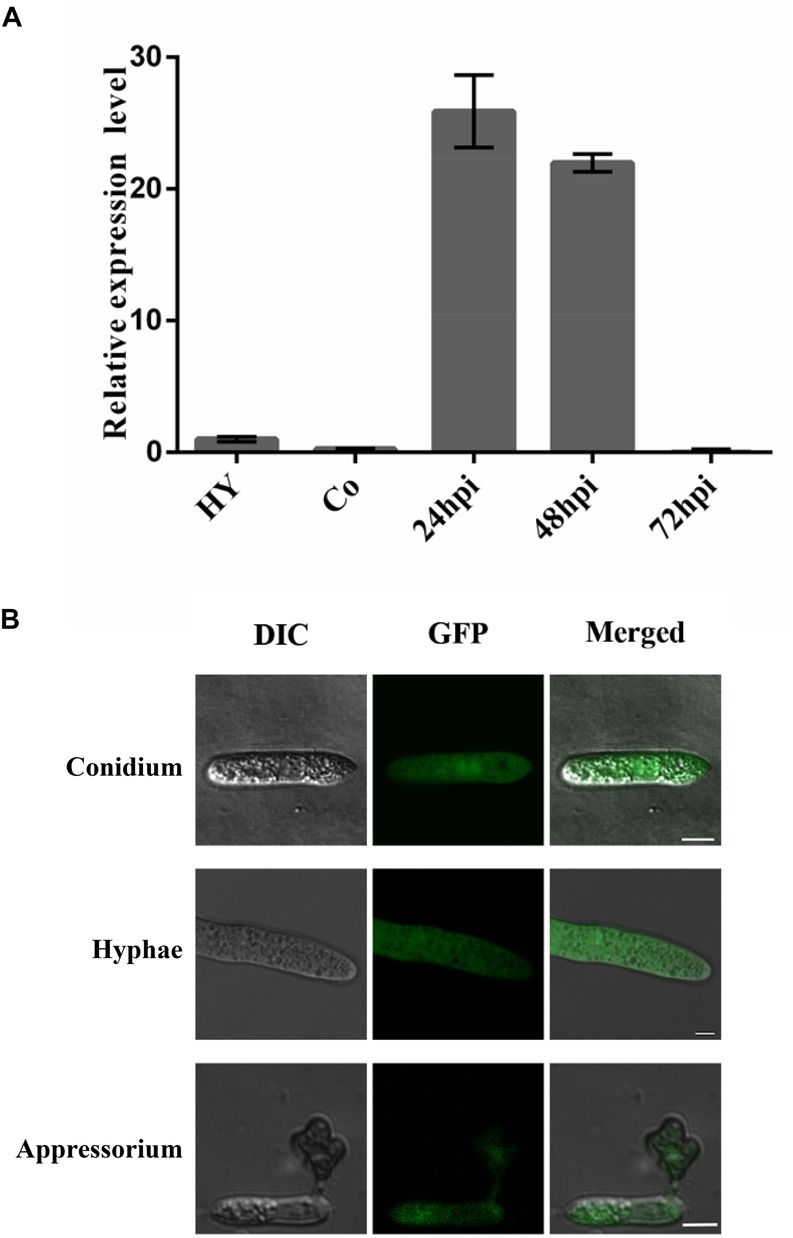
Relative expression and subcellular localization of *ChCDC25* at different stages of fungal development. **(A)** Expression profiles of *ChCDC25* relative to actin in conidia (Co) and hyphae (Hy) from PDB culture and at different infection stages in *Arabidopsis* (24, 48, and 72 hpi) by qRT-PCR. **(B)** Laser scanning confocal microscope (LSCM) observation of subcellular localization of *ChCDC*25:GFP. DIC, differential interference contrast; GFP, green fluorescent protein. Scale bar, 8 μm.

To gain insight into role of *ChCDC*25 in pathogenesis, gene expression profiles in vegetative hyphae, conidia, and infected *Arabidopsis* plants were examined using quantitative RT (qRT)–PCR. The results showed that the gene expression of *ChCDC*25 was relatively high in early infection stages (24 and 48 hpi) on *Arabidopsis*, suggesting that *ChCDC25* might be important in functions of appressorial formation and invasive hyphal growth (Figure 7A).

To assess subcellular localization, *ChCDC*25-GFP fusion constructs were generated and transformed into *ChCDC25-100.* The resulting transformant CΔ*ChCDC*25-100, which expressed *ChCDC*25-GFP, exhibited normal growth, conidiation, and virulence, indicating that the defects in the mutants were complemented. In GFP-tagged transformants, obvious GFP signals were found in the cytoplasm of vegetative hyphae, conidia, and appressoria (Figure 7B). These results indicated that *ChCDC*25 was constitutively expressed in the cytoplasm throughout the developmental stages. This was consistent with the gene expression profiles during developmental and infection stages.

### Yeast Two-Hybrid Assay to Identify ChCDC25 Interacting Partners

**FIGURE 8:**
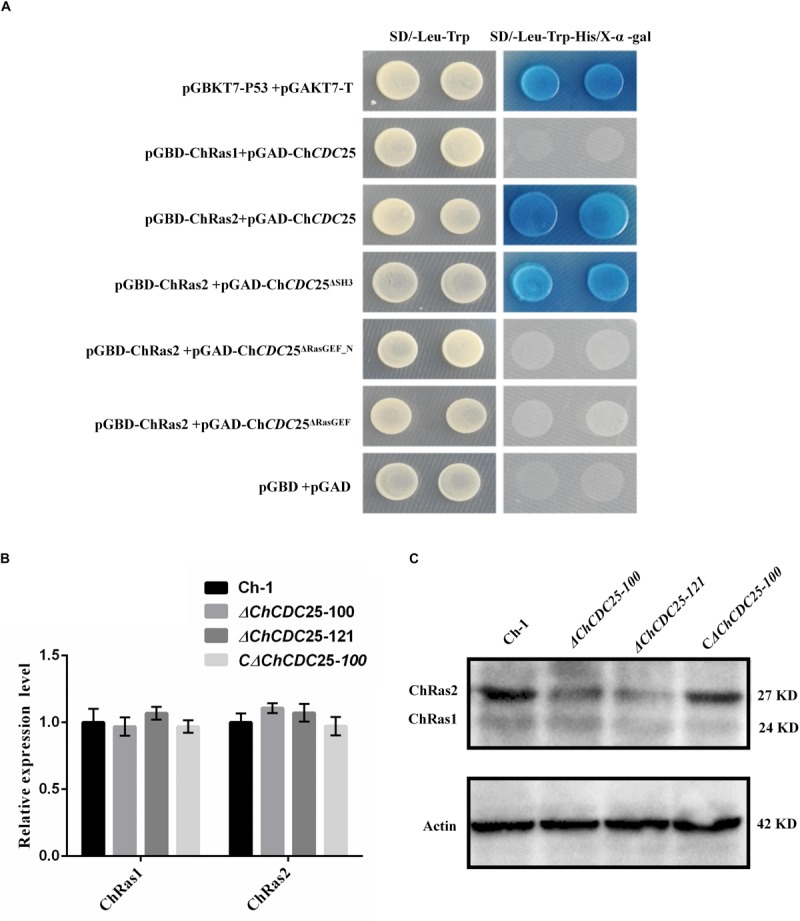
Yeast two-hybrid assay and Ras gene expression and protein levels in *ChCDC25* knockout mutants. **(A)** Yeast two-hybrid assay. Yeast two-hybrid screening was performed to determine putative physical interactions between *ChCDC*25 and Ras proteins and to further investigate potential interaction domains. Simultaneous cotransformation of the pGBK-Ras (bait-Ras1 or bait-Ras2) and pGAD-ChCDC25 (prey-ChCDC25, -ChCDC25^Δ^ ^*SH*3^, -ChCDC25^Δ^ ^*RasGEF_N*^, or -ChCDC25^Δ^ ^*RasGEF*^) vectors into the yeast two-hybrid gold strain resulted in activation of three reporter genes and growth on stringency media (-His/-Leu/-Trp/ + X-α-Gal). Yeast strains carrying pGBKT7-P53/pGADT7-T and pGBD/pGAD were used as positive and negative controls, respectively. **(B)** Transcript levels of Ras in *ChCDC25* knockout mutants. Quantitative PCR was performed using *C. higginsianum* actin as the endogenous standard. Each sample was analyzed in three independent biological duplications. **(C)** Ras protein levels in *ChCDC25* knockout mutants. Western blot analysis was conducted, and ChRas was detected using anti-KRas rabbit monoclonal antibody.

*ChCDC*25 was tentatively identified as a RasGEF protein, which prompted us to investigate its potential interactions. The protein sequence of Ras1 (CAA99298.1) and Ras2 (CAA95974.1) from *S. cerevisiae* was used to compare against the *C. higginsianum* genome database with BLASTp. The homologous proteins CH063_126029 (ChRas1) and CH063_07779 (ChRas2) were identified. Yeast two-hybrid analysis was carried out to test whether *ChCDC*25 can interact with Ras1 and Ras2. The vectors pGAD-ChCDC25, pGBD-ChRas1, and pGBD-ChRas2 were constructed. The results showed that ChCDC25 physically interacted with ChRas2, but not ChRas1 (Figure 8A).

To further clarify which domain of *ChCDC*25 may be involved in interactions with Ras2, we constructed three prey vectors pGAD-Ch*CDC*25^Δ^ ^*SH*3^, pGAD-Ch*CDC*25^Δ^ ^*RasGEF_N*^, and pGAD-Ch*CDC*25^Δ^ ^*RasGEF*^ lacking each respective domain. The resulting prey vector with bait vector pGBK-Ras2 was introduced in pairs into yeast strain Y2HGold, and the results indicated that ChCDC25 interacted with ChRas2 by the RasGEF_N and RasGEF domains (Figure 8A).

### Ras Protein Abundance Is Dependent on ChCDC25

To determine whether *ChCDC25* might regulate gene expression of *ChRas* by a feedback loop, we measured mRNA levels of *ChRas1* and *ChRas2* individually by qPCR between wild-type and *ChCDC25* deletion mutants. However, no significant differences between wild-type and *ChCDC25* deletion mutants in *ChRas1* or *ChRas2* gene expression were observed (Figure 8B). To further investigate ChRas protein expression, total protein of the wild-type, *ChCDC25* deletion mutants, and the complemented strain were tested using an anti-Ras antibody in Western blotting. Obviously, a significant decrease in ChRas2 protein abundance was found in the *ChCDC25* deletion mutant, whereas no appreciable change was found in ChRas1 protein abundance (Figure 8C). The results suggested that deletion of *ChCDC*25 affected Ras2 protein abundance in *C. higginsianum*.

## Discussion

In *S. cerevisiae*, the Cdc25/Ras/cAMP signal transduction pathway plays an important regulatory role in cell growth, differentiation, and proliferation (Robinson et al., 1987; Petitjean et al., 1990). However, the role of the CDC25 protein in phytopathogens is largely unexplored, especially for hemibiotrophic fungal pathogens. In this study, we found that *ChCDC25* is essential for mycelial growth, conidiation, conidial germination, appressorial formation, stress response, and pathogenicity in *C. higginsianum*. To our knowledge, this is the first report to reveal functions of *ChCDC25* in a hemibiotrophic phytopathogen and that Ras2 is the core target for the novel regulatory mechanism of *ChCDC25*.

Previous work showed that *CDC25* deletion mutants were more tolerant to oxidative and osmotic stresses in *S. cerevisiae* (Folch-Mallol et al., 2004). However, in *C. higginsianum*, the *ChCDC25* deletion mutants were more sensitive to cell wall and oxidative stresses and showed increased tolerance to salt stress. These results imply functional diversity of CDC25 among fungi.

In previous studies, the cAMP and MAPK signaling pathways were reported to regulate appressorial formation and pathogenicity in *C. higginsianum* (Wei et al., 2016; Yuan et al., 2016; Gu et al., 2017; Zhu et al., 2017). In this work, deletion of *ChCDC25* caused obvious defects in conidial germination and appressorial formation. The defects in surface recognition and appressorial differentiation suggested that *ChCDC*25 might be involved in the cAMP signaling pathway. Although exogenous applications of cAMP and IBMX could not rescue the shortened length of germ tubes, appressorial formation was elevated in the *ChCDC25* mutants. Furthermore, our results showed that the intracellular cAMP levels were reduced significantly in the ChCDC25 mutants. This indicated that *ChCDC*25 could regulate the formation of appressoria by affecting the content of cAMP in *C. higginsianum*. In addition, the pathogenicity assay showed that the appressoria formed by ChCDC25 mutants were non-functional, which led to lack of disease symptoms. Overall, these results suggested that *ChCDC*25-mediated cAMP signaling pathways regulate infection-related morphogenesis.

Subcellular localization in this study revealed that ChCDC25 is not located in the cytoplasmic membrane, but accumulates inside the cytoplasm. Although its structure and function suggest it should be localized the same as Ras protein, which is in the cytoplasm membrane, our localization results were consistent with previous reports from *Saccharomyces pombe* (Papadaki et al., 2002). In addition, analysis of the Cdc25 sequence revealed that the central uncharacterized region (residues 1122–1132) might contribute to nuclear localization. However, in *S. pombe*, Cdc25 is localized to the cytoplasm during interphase, and the protein moves into nucleus shortly before mitosis (Zeng and Piwnica-Worms, 1999). Thus, whether the protein localization of ChCDC25 alters during plant infection should be verified in future studies.

Previous studies have shown that *ChRgf*, encoding a GEF, is involved in regulating infection-related morphogenesis and pathogenicity of *C. higginsianum* (Gu et al., 2017). However, *ChRgf* is not required for invasive growth in wounded *Arabidopsis* leaves (Gu et al., 2017). However, ChCDC25 deletion mutants were defective in invasive growth in this study. Genetic and phylogenetic analyses indicated that ChCDC25 and ChRgf were not grouped together (Gu et al., 2017), suggesting that ChCDC25 and ChRgf might display distinct functions. In addition, ChCDC25 contains an SH3 domain, which is absent in ChRgf. Previous studies showed that SH3 domains of CDC25 are important for regulation of cytoskeleton and Ras pathways (Tratner et al., 1997; Tisi et al., 2008). For example, the SH3 domain of *S. cerevisiae* CDC25p binds to adenylyl cyclase and is hyperresponsive to Ras (Jones et al., 1991). Thus, the SH3 domain of ChCDC25 protein may be involved in various signaling pathways in *C. higginsianum*.

In *S. cerevisiae*, CDC25p as a guanine nucleotide protein regulated the RAS/adenylyl cyclase/protein kinase A pathway (Martegani et al., 1992). The Ras proteins are key elements for the regulation of cell growth and differentiation and act as molecular switches cycling between the active GTP-bound and inactive GDP-bound states (Bourne et al., 1991). In order to identify potential interacting partners of *ChCDC*25, yeast two-hybrid assay was carried out between *ChCDC*25 and Ras1/Ras2. The results showed that *ChCDC25* can interact only with Ras2, suggesting that Ras2 is the core target for the novel regulatory mechanism of ChCDC25. Furthermore, we found that *ChCDC*25 interacts with Ras2 by the RasGEF_N domain and the RasGEF domain. Because of the limitations of this test with the yeast two-hybrid assay, further research is needed to validate interactions of domains between ChCDC25 and Ras2. However, this result is in line with previous reports in yeast (Raaijmakers and Bos, 2009). In addition, it has been reported that the RasGEF domain is involved in the stabilization of CDC25 binding with Ras and that the RasGEF_N domain confers enhancement of catalytic activity to CDC25 for function as an upstream regulator of Ras (Raaijmakers and Bos, 2009).

In mammalian cell lines, *S. cerevisiae*, and *S. pombe*, deletion or genetic losses of CDC25 genes have similar effects as loss of the corresponding Ras genes (Mitin et al., 2005). In *S. cerevisiae*, both Ras1 and Ras2 regulate the activation of adenylyl cyclase (Powers et al., 1991; Hurwitz et al., 1995). In *M. oryzae*, the Ras2 protein plays an important role in regulating cAMP and the MAPK signaling pathways (Zhou et al., 2014). In *F. graminearum*, Ras2 is involved in hyphal growth, spore germination, and pathogenicity by regulating the Gpmk1 MAP kinase pathway (Bluhm et al., 2007). In *Colletotrichum trifolii*, dominant active mutants of Ras showed that Ras is involved in polarity, growth, and differentiation, by regulating the MAPK pathway (Chen and Dickman, 2004). In this study, a significant decrease in the abundance of the ChRas2 protein was found in the ChCDC25 mutant, whereas no appreciable change was found in abundance of the ChRas1 protein. The results suggested that *ChCDC*25 is required for stabilization of the Ras2 protein. Therefore, *ChCDC*25 may regulate Ras signaling pathways, which in turn affects a variety of downstream signaling pathways. Furthermore, some studies have shown that Cdc25 was phosphorylated by PKA subunits, and Kss1 and Fus3 MAPK kinases to activate the meiotic G2/M transition (Cherkasova et al., 2003; Deminoff et al., 2006). Further work on the role of *ChCDC*25 for MAPKs cross-regulation of Ras signaling is needed.

## Conclusion

In summary, our results suggest that *ChCDC*25 interacts with Ras2 to integrate multiple distinct signals for multifaceted functions in development and virulence in *C. higginsianum.*

## Data Availability Statement

The raw data supporting the conclusions of this article will be made available by the authors, without undue reservation, to any qualified researcher.

## Author Contributions

LZ and YY designed the experiments. YY, JT, and QY performed the experimental work. YY, LZ, QG, HL, and JH analyzed the data. YY, LZ, and TH wrote the manuscript.

## Conflict of Interest

The authors declare that the research was conducted in the absence of any commercial or financial relationships that could be construed as a potential conflict of interest.

## Footnotes

1 http://fungi.ensembl.org/Colletotrichum_higginsianum/Info/Index

2 http://smart.embl-heidelberg.de/
