# Supplementary material for: ChCDC25 Regulates Infection-Related Morphogenesis and Pathogenicity of the Crucifer Anthracnose Fungus Colletotrichum higginsianum
Source: Front Microbiol. 2020 May 8;11:763. doi: 10.3389/fmicb.2020.00763 (PMC7227425; doi:10.3389/fmicb.2020.00763)
Supplement: Supplementary file 1 [file Table_1.doc]

**Supplementary material**

**Table S1: Primers used in this study.**

| **Primer name** | **Sequence (5’- 3’)** | **Usage** |
| --- | --- | --- |
| *ChCDC*25F1F | GGGGTACCCCATTAAGATCAGGTTGAAT | PCR amplification for *UvPmk1*  flanking sequences |
| *ChCDC*25F1R | GCTCTAGAAGCTGTGTCGTCCACGCATC |
| *ChCDC*25F2F | ACGCGTCGACCCAGTATGGTGGGCTGGCTG |
| *ChCDC*25F2R | CCCAAGCTTCCCCCTGTTTCCCCGTTCCT |
| *ChCDC*25comF | AACTGCAGTCGCTCTCATAAACAAAGG | PCR amplification for *UvPmk1*  complemrntal fragment |
| *ChCDC*25comR | AACTGCAGTCATAGGAAGCCAGATTCGGC |
| *ChCDC*25ProbeF | TCAGTTACGACAGCACATTAGG | PCR amplification for probe |
| *ChCDC*25ProbeR | GGCAGTCTGGTTGTTGAGTC |
| *ChCDC*25F | TGTTACGAGCACCGATGAGA | RT-PCR |
| *ChCDC*25R | TTGTAGTAGAAGAGGCGACCAT |
| qRT- *ChCDC*25F | GCAGCAGCATGTCGATTCC | qRT-PCR |
| qRT- *ChCDC*25R | GGTGAATGATTGCGAGGTGAT |
| qRT- *ChRas*1F | AATCCTGCTTGACCATCCAATT | qRT-PCR |
| qRT- *ChRas*1F | AACCCTCTCCCGTCCTCAT |
| qRT- *ChRas*2F | GGGAAGACCGCTTTGACGAT |
| qRT- *ChRas*2R | CCGAAGGGCTGTGTACTCTT |
| pGAD- *ChCDC*25-SP | gtgggcatcgatacgggatccATGCTGATGACACAGACCGCA | PCR amplification for pGAD- *ChCDC*25 fragment |
| pGAD- *ChCDC*25-SP | cagctcgagctcgatggatccTCATAGGAAGCCAGATTCGGC |
| pGBD-ChRas1-SP | aggccgaattcccggggatccATGGCGTCCAAGTTTCTGAGG | PCR amplification for pGBD-ChRas1 fragment |
| pGBD-ChRas1-AP | ccgctgcaggtcgacggatccTCACATTAGTACGCACTTGGAGCA |
| pGBD-ChRas2-SP | aggccgaattcccggggatccATGGCGGGAAAAATGGTGC | PCR amplification for pGBD-ChRas2 fragment |
| pGBD-ChRas2-AP | ccgctgcaggtcgacggatccTCACAAGACGACGCATTTACTCTT |
| pGAD-Ch*CDC*25^ΔSH3^F1 | ACTTCAAGTCCTCGTTTCCCA | PCR amplification for pGAD-Ch*CDC*25^ΔSH3^ fragment |
| pGAD-Ch*CDC*25^ΔSH3^R1 | CAACTTCTTCCTGCTGCTCGG |
| pGAD-Ch*CDC*25^ΔSH3^F2 | ggcacaAGCACCGATGAGATCCCCG |
| pGAD-Ch*CDC*25^ΔSH3^R2 | cagctcgagctcgatggatccTCATAGGAAGCCAGATTCGGC |
| pGAD-Ch*CDC*25^ΔRasGEF_N^F1 | gtgggcatcgatacgggatccATGCTGATGACACAGACCGCA | PCR amplification for pGAD-Ch*CDC*25^ΔRasGEF_N^ fragment |
| pGAD-Ch*CDC*25^ΔRasGEF_N^R1 | gccTGGCGCAAGCTTGTTATCCC |
| pGAD-Ch*CDC*25^ΔRasGEF_N^F2 | ataacaagcttgcgccaGGCCAGCAAGCCGGAGCC |
| pGAD-Ch*CDC*25^ΔRasGEF_N^R2 | cagctcgagctcgatggatccTCATAGGAAGCCAGATTCGGC |
| pGAD-Ch*CDC*25^ΔRasGEF^F1 | gtgggcatcgatacgggatccATGCTGATGACACAGACCGCA | PCR amplification for pGAD-Ch*CDC*25^ΔRasGEF^ fragment |
| pGAD-Ch*CDC*25^ΔRasGEF^R1 | cgtcttctcgTTTGAGCTTCTTCATGTTCTTGGG |
| pGAD-Ch*CDC*25^ΔRasGEF^F2 | gaagctcaaaCGAGAAGACGAAAAGATCGTGAG |
| pGAD-Ch*CDC*25^ΔRasGEF^R2 | cagctcgagctcgatggatccTCATAGGAAGCCAGATTCGGC |
| β-tubulin-F | AGAAAGCCTTGCGACGGAACA | Reference for RT-PCR and qRT-PCR |
| β-tubulin-R | CCTCCAGGGTTTCCAGATTA |
